# Supplementary material for: Altered microRNA Transcriptome in Cultured Human Liver Cells upon Infection with Ebola Virus
Source: Int J Mol Sci. 2021 Apr 6;22(7):3792. doi: 10.3390/ijms22073792 (PMC8038836; doi:10.3390/ijms22073792)
Supplement: Supplementary file 1 [file ijms-22-03792-s001.zip › Supplementary_File/C_ GO_Analysis_Results/16-30nt_go_Reston-96h-Huh7_vs_Control-96h-Huh7_up.mature_mirna_targets/MF_GeneRatioDotPlot.pdf]

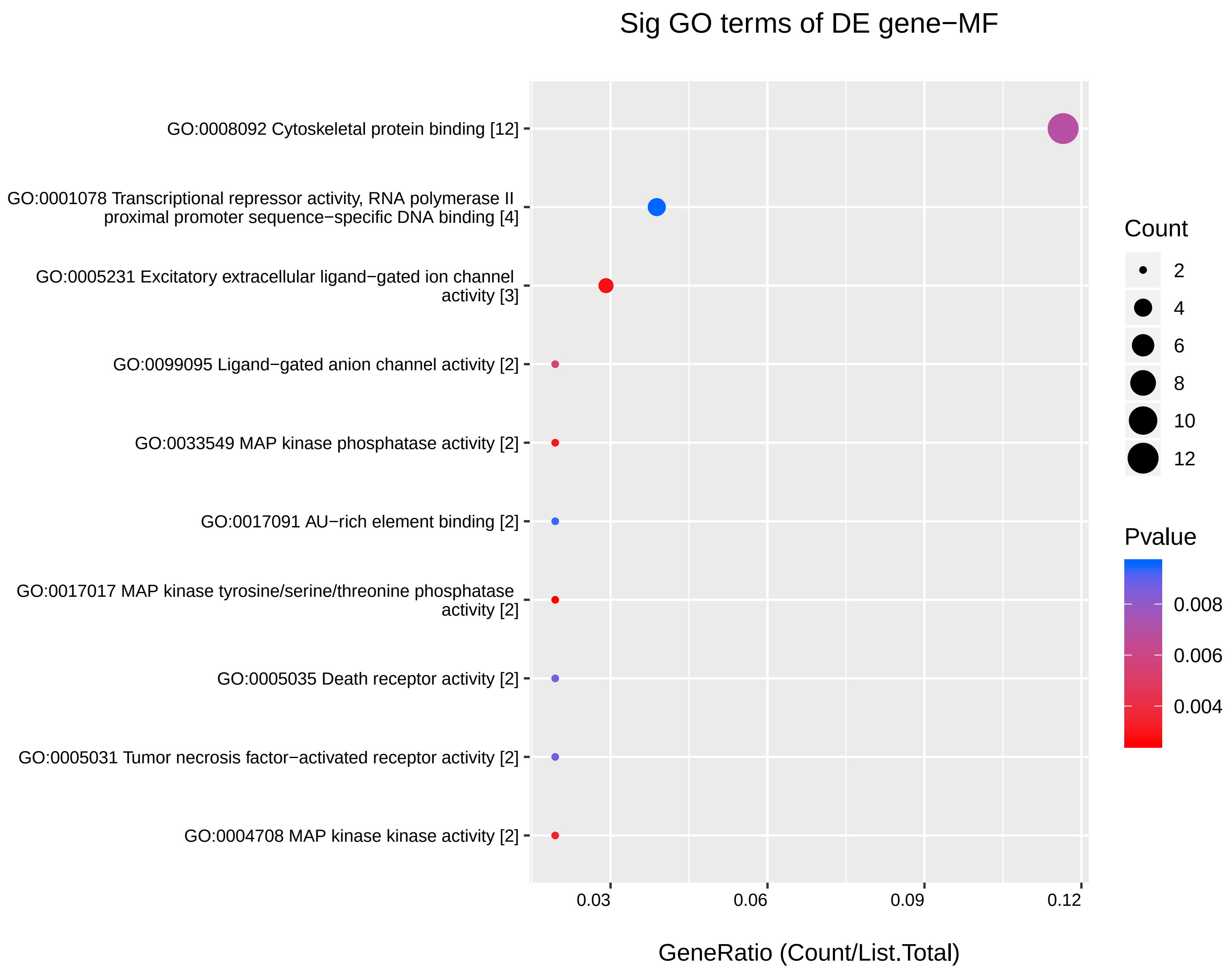
Sig GO terms of DE gene−MF
GO:0008092 Cytoskeletal protein binding [12]
GO:0001078 Transcriptional repressor activity, RNA polymerase II proximal promoter sequence−specific DNA binding [4]
GO:0005231 Excitatory extracellular ligand−gated ion channel activity [3]
GO:0099095 Ligand−gated anion channel activity [2]
GO:0033549 MAP kinase phosphatase activity [2]
GO:0017091 AU−rich element binding [2]
GO:0017017 MAP kinase tyrosine/serine/threonine phosphatase activity [2]
GO:0005035 Death receptor activity [2]
GO:0005031 Tumor necrosis factor−activated receptor activity [2]
GO:0004708 MAP kinase kinase activity [2]
0.03
0.06
0.09
0.12
GeneRatio (Count/List.Total)
Count
2
4
6
8
10
12
Pvalue
0.008
0.006
0.004
